# Expression, Purification and Characterization of Arginase from *Helicobacter pylori* in Its Apo Form

**DOI:** 10.1371/journal.pone.0026205

**Published:** 2011-10-20

**Authors:** Jinyong Zhang, Xiaoli Zhang, Chao Wu, Dongshui Lu, Gang Guo, Xuhu Mao, Ying Zhang, Da-Cheng Wang, Defeng Li, Quanming Zou

**Affiliations:** 1 Department of Clinical Microbiology and Immunology, College of Medical Laboratory, Third Military Medical University, Chongqing, China; 2 National Laboratory of Biomacromolecules, Institute of Biophysics, Chinese Academy of Sciences, Beijing, China; University of South Florida College of Medicine, United States of America

## Abstract

Arginase, a manganese-dependent enzyme that widely distributed in almost all creatures, is a urea cycle enzyme that catalyzes the hydrolysis of L-arginine to generate L-ornithine and urea. Compared with the well-studied arginases from animals and yeast, only a few eubacterial arginases have been characterized, such as those from *H. pylori* and *B. anthracis.* However, these enzymes used for arginase activity assay were all expressed with LB medium, as low concentration of Mn^2+^ was detectable in the medium, protein obtained were partially Mn^2+^ bonded, which may affect the results of arginase activity assay. In the present study, *H. pylori* arginase (RocF) was expressed in a Mn^2+^ and Co^2+^ free minimal medium, the resulting protein was purified through affinity and gel filtration chromatography and the apo-form of RocF was confirmed by flame photometry analysis. Gel filtration indicates that the enzyme exists as monomer in solution, which was unique as compared with homologous enzymes. Arginase activity assay revealed that apo-RocF had an acidic pH optimum of 6.4 and exhibited metal preference of Co^2+^>Ni^2+^>Mn^2+^. We also confirmed that heat-activation and reducing regents have significant impact on arginase activity of RocF, and inhibits S-(2-boronoethyl)-L-Cysteine (BEC) and Nω-hydroxy-nor-Arginine (nor-NOHA) inhibit the activity of RocF in a dose-dependent manner.

## Introduction

*Helicobacter pylori* (*H. pylori*), a gastric pathogen that infects more than 50% of the world population, is the cause of gastritis and peptic ulcers and a risk factor for gastric cancer [1], [2]. It usually causes chronic infection and persists for the life of the host and is not eradicated despite a vigorous immune response was raised by host immune system [3], suggesting that the bacterial reserve some mechanisms for escaping from host immune response, such mechanisms have been under extensive study and reported elsewhere [4], [5], [6].

Arginase is a urea-cycle enzyme that catalyzes the hydrolysis of L-arginine to yield L-ornithine and urea, and it is present in almost all known life forms and plays a crucial role in nitrogen metabolism [7], [8]. In the gastric pathogen *H. pylori*, arginase is encoded by *rocF* gene and the resulting protein RocF exhibits extensive genotypic and phenotypic variation [9], [10]. Recently, RocF was identified to show some immunosuppressant properties and is probably involved in the bacterium’s evasion of the host’s immune system [11]. Firstly, RocF competes with host inducible nitric oxide synthase for the common substrate L-arginine, thus reduces the synthesis of NO, an important component of innate immunity and an effective antimicrobial agent that is able to kill the invading pathogens directly. Research indicates that arginase-deficient bacteria are more sensitive to NO-dependent killing by host macrophages, whereas the wild-type bacteria exhibited no loss of survival [12], [13]. Secondly, RocF is involved in inhibiting human T cell proliferation and T cell CD3ζ expression, and thus efficiently reduces host cellular immune response by contributing to the inability of T cells. [14]. Finally, the product urea is utilized by urease to generate ammonia and carbon dioxide, which helps to neutralize the acidic environment of the stomach and thus facilitate colonization by the bacterium [15]. These clue that RocF could inhibit host innate defense and adaptive immune response simultaneously to facilitate the pathogenesis of *H. pylori*, thus RocF is considered to be a new virulence of *H. pylori* [11].

The arginase activity of RocF has been characterized by McGee *et al* previously by using the enzyme expressed in LB medium and purified by Ni-NTA affinity chromatography, the enzyme exhibit an metal preference of Co^2+^>Mn^2+^>Ni^2+^ and show optimal activity at pH 6.1 [16]. They also noticed that the specific activity of purified arginase varied in different elutions, owing to the partial inhibition of enzyme activity at high concentration of imidazole. Besides, in order to determine the crystal structure of RocF, we have also expressed the protein in LB medium and purified it by Ni-NTA affinity and gel filtration chromatography [17], the resulting protein was partially Mn^2+^ bounded as determined by flame photometry analysis, the extra divalent ions would also affect the determination of arginase activity of RocF. In order to minimize the influence of imidazole and the extra Mn^2+^ on arginase activity assay, apo-RocF is required.

In this paper, we have developed a Mn^2+^ and Co^2+^ free minimum medium to express apo-form of RocF, the resulting protein was first purified by Ni-NTA affinity chromatography and then by gel filtration chromatography to exclude imidazole from the enzyme, Co^2+^, Mn^2+^ and Ni^2+^ were not detected in the protein as determined by flame photometry analysis, indicating apo-RocF was obtained, the protein was used to determine the optimum of divalent ions and pH value; the impact of heat-activation, reducing agents and potent inhibitors on arginase activity of apo-RocF were also studied.

## Results and Discussion

### Protein expression and purification

A minimum medium was chosen for the expression of apo-RocF, the formation of the medium was listed in Table 1, Mn^2+^ and Co^2+^ were excluded of the medium as they were critical for arginase activity [16], [18], [19], [20], other metal ions such as Fe^2+^ and Mg^2+^ that essential for bacterial normal growth were retained in the medium. RocF was expressed at 37°C under the induction of 1 mM IPTG, SDS-PAGE of cell lysates showed a major protein band of the expected 37 kDa size. RocF was expressed in the soluble fraction and the level was about 10% of the total cellular proteins, which was less than 20% of RocF expressed in LB medium (data unreported). By virtue of the His6 tag at the C-terminal of the protein, the sonicated supernatant was loaded onto Ni-NTA column directly for affinity chromatography, SDS-PAGE indicate that the eluate had a purity of about 90%, the eluate was then concentrated and applied to gel filtration chromatography, fractions corresponding to RocF monomer was pooled and analyzed by SDS-PAGE, the purity was up to about 95%. The protein was found to be highly unstable and will lost considerable of its activity quickly, which was in consistence with the previous report by McGee *et al* [16], the purified protein was stored at 4°C and used for activity assay within 3 days to minimize lose of its activity in the present study.

**Table 1:** The formation of the minimum medium used for expression of apo-RocF.

Regents	Concentration	Regents	Concentration
Na2HPO4	8 g	CaCl2	0.3 mM
KH2PO4	4 g	FeCl3	0.03 mM
Glucose	4 g	H3BO3	0.03 mM
NH4Cl	0.5 g	CuCl2	0.03 mM
NaCl	0.5 g	Biotin	1 mg
MgSO4	1 mM	Thiamin	1 mg

### Oligomerization of apo-RocF

**Figure 1:**
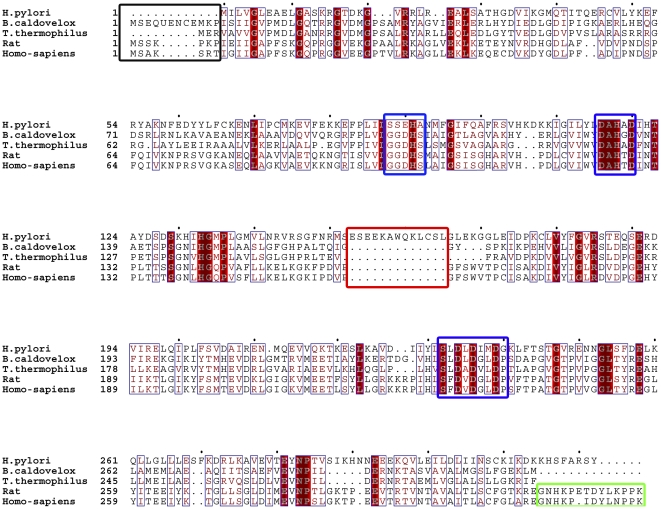
Alignment of amino acid sequences of RocF with homologous enzymes. Colored outlines indicate identical and similar amino acid residues, respectively. Residues in the three blue boxes represent the three conserved domain that consider being important for ions binding and the catalytic process. Residues in the red box were an insertion that only observed in RocF but not other arginases. Residues in the black box revealed the difference in the N-terminal of aligned enzymes. Residues in the green box represent the domain termed ‘oligomerization motif’ that exist in eukaryotic arginases, which was absent in *T. thermophilus* and *B. caldovelox* arginases but was substituted by a non-conserved motif in RocF.

Gel filtration indicates that apo-RocF exists as monomer in solution (Figure S1), which is in consistent with the former report that RocF exists as a mixture of monomer and dimer with monomer being the major form, and dimmer was undetectable with salt concentrations higher than 100 mM [21]. This is strange because only two different oligomeric states of arginase have been reported so far. In general, the eukaryotic arginases tend to be trimeric whereas the bacterial ones tend to be hexameric [22], [23]. To explain the reason for the difference, the amino acid sequences of several arginases including those from rat [24], homo-sapiens [22], [23], *T. thermophilus*, *B. caldovelox* [22], [23]and *H. pylori* were aligned with the online program ClusterW2 (http://www.ebi.ac.uk/Tools/msa/clustalw2/), the structures of these arginases except RocF were available at PDB. As shown in Figure 1, RocF retains the three conserved characteristic domains including GGDHS, SXDXDXDP and DAHXD that are critical for ions binding and the catalytic process as other arginases [22], [23]. However, an insertion of 13 residues (ESEEKAWQKLCSL) was observed in the middle of the sequence of RocF, the function of this domain was not clear for the moment. Besides, RocF exhibits considerable differences in N and C-terminal sites and the C-terminal sequence were thought to be essential for the oligomerization of arginase. In brief, the C-terminal 14 residues in eukaryotic arginases (Figure 1, green box) termed an ‘oligomerization motif’ was essential for trimer formation [22], [23]. Sequences alignment indicates that this motif was absent in prokaryotes arginases, on the other hand, prokaryotes arginases form hexamer by interactions of residues from the C-terminal through salt bridges and hydrogen bonds [22], [23]. However, RocF was unique among all of these enzymes, it adopts a "KHSFARSY" motif in the C-terminal which was not found in other prokaryotes arginases and shows no similarity with the ‘oligomerization motif’, we propose that this motif may responsible for the oligomeric organization of RocF as monomer.

### Protein determinations

Researches revealed that arginase is a binuclear manganese metalloenzyme, each monomer was able to bind two manganeses [22], [23], however, some arginases can be activated by other divalent metal ions such as cobalt and nickel, particularly, it was reported that arginase from *H. pylori* and *B. anthracis* exhibit optimal catalytic activity at the presence of Co^2+^ and Ni^2+^, respectively [16], [20], so we assume that the three dimensional structure of RocF in complex with Co^2+^ may undergo conformation switch as compared with RocF in complex with Mn^2+^. In order to gain Co^2+^ bounded RocF for structure studies, we have tried to purify RocF expressed in LB medium by Co-NTA affinity chromatography, however, the resulting protein was both Co^2+^ and Mn^2+^ bounded as determined by flame photometry analysis (Table 2) , while Ni^2+^ was undetectable. The ratio was 8∶1 for RocF and Co^2+^ and 3.6∶1 for RocF and Mn^2+^, indicating that the affinity between RocF and Mn^2+^ was relatively higher than Co^2+^ and Ni^2+^. Based on these results, we propose that RocF was Mn^2+^ bounded *in vivo* as other arginases. When the protein was purified by Ni-NTA affinity chromatography, only Mn^2+^ was detected in the protein with a ratio of 1∶1.8 (Table 2), these results revealed that protein expressed in LB medium was already Mn^2+^ bounded and this will affect the results for arginase activity assay. The ratio between Mn^2+^ and RocF was nearly 1∶2, obviously lower than the saturation value of 2∶1. These results indicate that these metal ions bind RocF with low affinity, which was supported by the finding that arginases will lose divalent metal ions during the process of dialysis [25].

**Table 2:** Flame photometry analysis of divalent metal ions in RocF obtained in different medium and purified by different methods.

Medium	Method for purification	RocF (µM)	Co^2+^ (µM)	RocF: Co^2+^	Ni^2+^ (µM)	RocF: Ni^2+^	Mn^2+^ (µM)	RocF: Mn^2+^
LB	Co-NTA and gel filtration	146	18	8∶1	<1	-	41	3.6∶1
LB	Ni-NTA and gel filtration	122	<1	-	<1	-	66	1.8∶1
Minimum	Ni-NTA and gel filtration	74	<1	-	<1	-	<1	-

In order to obtain apo-RocF for arginase activity assay, we developed a Mn^2+^ and Co^2+^ free minimum medium to express RocF and the resulting protein was purified by Ni-NTA affinity and gel filtration chromatography, these three kinds of metal ions in the protein were also determined, as shown in table 2, the concentration of these metal ions were not detectable as compared with RocF, indicating that apo-RocF was obtained and this form of the enzyme was used for arginase activity assay. Besides, imidazole also has considerable impact on the colorimetric development by L-ornithine. In this study, the protein eluated from Ni-NTA was also used for arginase activity assay, however, after the addition of 250 µl of ninhydrin (4 mg/ml) at 95°C, the characteristic yellow color developed within a few minutes but diminished after 1h, indicating that imidazole should be excluded from the protein for accurate activity assay. In this study, gel filtration chromatography was applied for further purification of apo-RocF as well as desalting.

The concentration of the purified protein was determined by BCA assay (Thermo Scientific PIERCE®) [26] according to the protocol and adjusted to 0.05 mg ml^−1^ for arginase activity assay. A standard curve was generated and used to determine the concentration of L-ornithine in the reaction mixture, the R^2^ value was 0.9989 (Figure S2).

### Optimal metal ions and pH of apo-RocF

**Figure 2:**
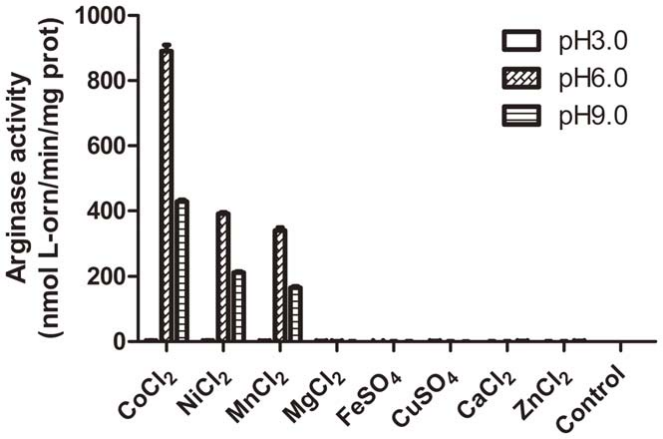
Apo-RocF exhibits optimal catalytic activity at the existence of cobalt in an acidic pH. Apo-RocF was first heat-activated at the existence of different divalent metal ions for 30 min at 55°C and the arginase activity was measured at pH 3.0, 6.0 and 9.0 as described in methods. Arginase activity was detectable when assayed with CoCl_2_, MnCl_2_ and NiCl_2_ at pH 6.0 and 9.0, the highest activity was observed at the present of Co^2+^ at pH 6.0.

The activity of the purified apo-RocF was measured at the presence of different divalent metal ions at pH 3.0, 6.0 and 9.0, as shown in Figure 2, unlike the well characterized mammalian arginases, apo-RocF show optimal catalytic activity when heat-activated with Co^2+^, significantly higher than incubated with Ni^2+^ and Mn^2+^, the specific activity was slightly higher when heat-activated with Ni^2+^ as compared with Mn^2+^. No arginase activity was detected at the presence of other divalent ions. Obviously, the highest activity appears at pH 6.0, which was in consistence with the report that RocF had an acidic pH optimum [16]. Our data suggest that apo-RocF show its metal preference of Co^2+^>Ni^2+^>Mn^2+^, which was different form the report of Co^2+^>Mn^2+^>Ni^2+^ for Mn^2+^ partially bounded RocF [16]. Apo-RocF also exhibit considerable arginase activity at pH 9.0, which was half as that at pH 6.0, no activity was detected at pH 3.0, probably due to denature of the enzyme under acidic conditions. The fresh apo-RocF yield a high arginase activity of nearly 900U, which was nearly the same as the activity of *B. anthracis* arginase [20] but was 100 times higher than RocF reported previously [16], indicating the extra imidazole and Mn^2+^ have significantly impact on arginase activity of RocF.

**Figure 3:**
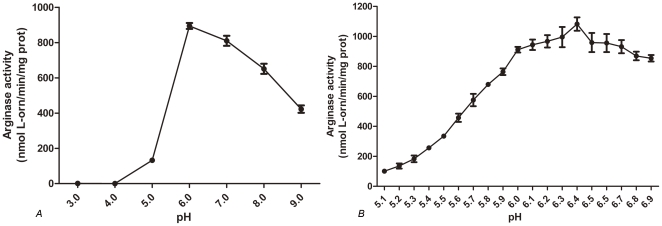
Apo-RocF shows optimal catalytic activity at pH 6.4. Apo-RocF was heat-activated with CoCl_2_ at 55°C and then assayed for arginase activity at 37°C for 60 min in arginase buffer at various pH values. Data are presented as mean arginase activity ± standard deviation. A) Screen of arginase activity of apo-RocF between pH3.0 to 9.0. B) Further Screen of arginase activity of apo-RocF between pH 5.1 to 7.9.

RocF was considered to be important for survival of *H. pylori* in the acidic gastric environment [27]. In the present study, by determination of arginase activity of apo-RocF at various pH value revealed that it exhibit a pH optimum of 6.4 (Figure 3), which was nearly the same as the report of 6.1 [16], this is unique because all other arginases characterized have a alkaline pH optimum of 9 to 11 [20], [28], so we consider that the acid pH optimum of RocF may be an adaptation to the acidic colonization environment of *H. pylori*.

### Heat-activation was essential for arginase activity of apo-RocF

**Figure 4:**
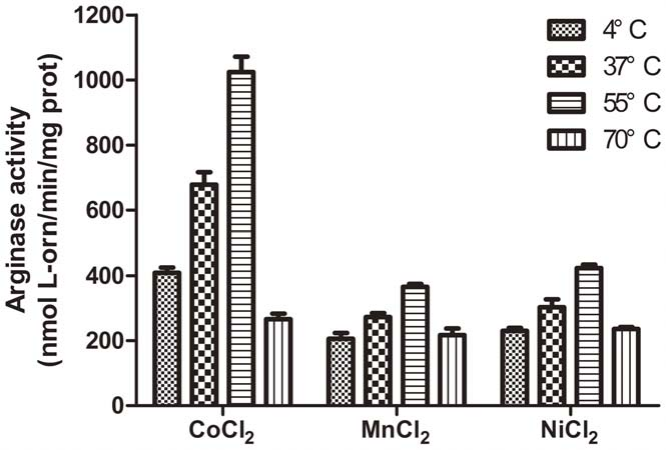
Effect of heat-activation on arginase activity of apo-RocF. Apo-RocF was heat- activated at 37°C, 55°C and 70°C or maintained on ice for 30 minutes at the presence of 5 mM CoCl_2_, MnCl_2_ or NiCl_2_, and then assayed with arginase buffer at pH 6.4 (buffered by MES). Data are presented as mean arginase activity ± standard deviation.

Previous studies indicated that arginase activity significantly increased after head-activation with metal ions at 55°C before reaction [16], [20]. In this study, apo-RocF was incubated with CoCl_2_, MnCl_2_ and NiCl_2_ at 4°C, 37°C, 55°C and 70°C for 30 min and then used for activity assay. Remarkably, heat treatment dramatically increased arginase activity in the presence of these ions. When heat-activated at the existence of Co^2+^, the activity at 55°C was 2.5, 1.5 and 3 times higher than heat-activated at 4°C, 37°C and 70°C, respectively. Similar results were observed when heat-activated with Mn^2+^ and Ni^2+^ but the difference was not so obviously (Figure 4). These results indicate heat-activation is essential for arginase activity, probably by promoting the binding of RocF with these ions. Lower activity was observed at 70°C, maybe the enzyme is not stable as before or even denatured at such a high temperature, thus resulting in a lower activity.

### Reducing agents significantly reduce arginase activity of apo-RocF

**Figure 5:**
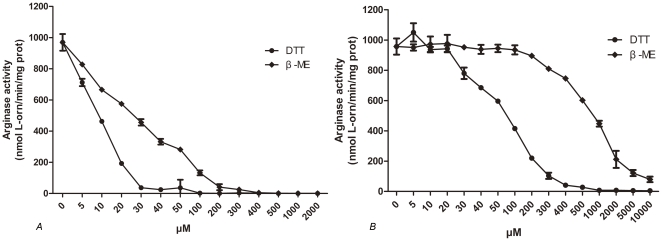
Apo-RocF is extremely sensitive to reducing agents. Various concentrations of reducing agents DTT or β-ME were added into the reaction mixture before (A) or after (B) heat-activation with CoCl_2_. Reducing agents were replaced with sterile water in control tubes. The 50% inhibitory concentration (IC50) was determined from the graph.

Previous studies revealed that RocF is extremely sensitive to reducing agents such as dithiothreitol (DTT) and 2-mercaptoethanol (β-ME) and its activity could be inhibited by small dose of these agents [16], [29]. In this study, the impact of DTT and β-ME on the activity of apo-RocF were also evaluated, various concentration of these agents were added into the reaction mixture before or after heat-activation at 55°C at the existence of Co^2+^, as shown in Figure 5, both DTT and β-ME have significant influence on its activity. Obviously, RocF is more sensitive to DTT than β-ME, and reducing agents will resulting in lost more of arginase activity when added into the reaction mixture before heat-activation. When reducing agents were added before heat-activation, the arginase activity of apo-RocF reduced dramatically even the concentration of reducing agents was 5 µM, and arginase activity reduced quickly as the concentration of reducing agents increase, 30 µM of DTT and 300 µM of β-ME will resulted in completely loss of arginase activity, with 50% inhibitory concentrations (IC50) of 10 µM and 30 µM, respectively. However, when added after heat-activation, the activity of apo-RocF was not affected even the concentration of DTT reached 20 µM, and 1 mM DTT is required for completely lost of arginase activity, which was 30 times higher than added before incubation. For β-ME, the activity began to reduce when the concentration reached 100 µM, and the activity was still detectable even the concentration reached 10 mM. Similar results were observed at the present of Mn^2+^ and Ni^2+^ (data unreported). Compared with the results reported previously, our results indicate that apo-RocF was more sensitive to reducing agents. These difference further confirmed that heat-activation at 55°C will significantly promote the binding of metal ions with RocF.

As there are six cysteines in the amino acid sequence of RocF, it was believed that disulphide bonds are critical for the catalysis process and essential for the activity of the enzyme, and the existence of a disulphide bond between Cys66 and Cys73 was confirmed by mass spectrometric studies [30], so it was suggested that reducing agents reducing the activity of RocF probably by disturbing the formation of disulphide bonds. However, mutational analysis shows that disulphide bonds are not important for catalytic activity [29]. Further more, we have determined the three dimensional structure of RocF and no disulphide bonds were observed between these cysteines (to be published). Besides, we have noticed that precipitation appeared as soon as these reducing agents were added into apo-RocF. These results indicate that no disulphide bonds was exist in RocF, and reducing agents significantly reducing the activity probably by competes with the enzyme for metal ions, as the later were important for the stability and enzymatic catalytic process of RocF, this assumption was supported by a former report [29].

### BEC and nor-NOHA inhibit arginase activity of apo-RocF in a dose-dependent manner

**Figure 6:**
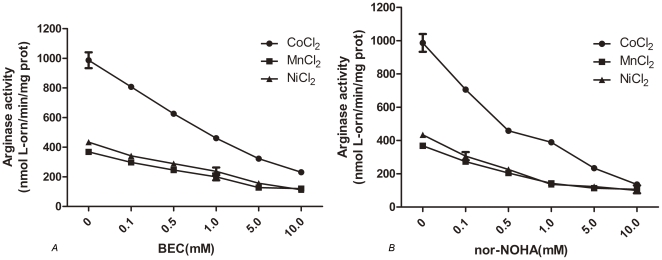
Inhibitors BEC (A) and nor-NOHA (B) inhibit argianse activity of apo-RocF in a dose-dependent manner. Apo-RocF was first heat-activated at 55°C at the existence of CoCl_2_, MnCl_2_, or NiCl_2_, arginase activity was then assay with arginase buffer at pH 6.4 contain different concentrations of inhibitors. Inhibitors were replaced with sterile water in control tubes. The 50% inhibitory concentration (IC50) was determined from the graph.

BEC and nor-NOHA are two L-arginine analogues that have successfully co-crystallized with arginases from various species [31], [32], [33], indicating that these chemicals may server as potent inhibitors and are important candidates for novel anti-bacterial drug development. In this study, the inhibitory effect of the two chemicals on arginase activity of apo-RocF was assayed, as shown in Figure 6, both BEC and nor-NOHA are able to inhibit RocF activity in a dose-dependent manner. Notably, the highest inhibitory effect was observed when heat- activated with Co^2+^, with 50% inhibitory concentrations (IC50) of 1 mM and 0.5 mM for BEC and nor-NOHA, respectively, indicating that nor-NOHA exhibit a higher affinity with RocF. However, the inhibitory effect of these inhibitors was not significant as compared with reducing agents, when the concentration of these chemicals reached 10 mM, the specific activity was still detectable. Similar results were observed when heat-activated with Mn^2+^ and Ni^2+^. Interestingly, the inhibitory effect of the two chemicals on apo-RocF activity can be divided into two parts, when the concentration of BEC were lower than 5 mM, the activity of RocF decreased quickly as the concentration of BEC increase; however, when the concentration of BEC were higher than 5 mM, the activity of RocF decreased in a lower speed. For nor-NOHA, the corresponding concentration was 0.5 mM, obviously lower than BEC. These results indicate that nor-NOHA and its analogues are more effective to serve as potent inhibitors for RocF than BEC.

## Materials and Methods

### Cloning and expression of apo-RocF

The *rocF* gene (NCBI-Gene ID: 899897) encoding *H. pylori* arginase RocF was amplified by PCR from *H. pylori* strain 26695 genomic DNA and cloned *via* introduced *NdeI/XhoI* restriction sites into the pET22b vector (Novagen) resulting in the recombinant plasmid pET22b-*rocF* as described previously [17]. Apo-RocF was obtained by expression of RocF in a Mn^2+^ and Co^2+^ free minimal medium. In brief, *E. coli* strain BL21 (DE3) (Novagen) competent cells transformed with the recombinant plasmid pET22b-*rocF* were first grown in LB medium containing 100 mg ml^−1^ ampicillin at 37°C until the OD_600_ of the culture reached 0.8. The cells were harvested by low-speed centrifugation, resuspended and washed twice in minimal medium, cells was then transferred to minimal medium containing 100 mg ml^−1^ ampicillin and incubated for 2 h at 37°C, isopropyl β-D-1-thiogalactopyranoside (IPTG) was then added to the culture at a final concentration of 1 mM to induce the expression of the recombinant protein at 37°C for 3 h. Cells was harvested by centrifugation at 4,000 rpm for 30 min at 4°C.

### Purification and determination of apo-RocF

Cell pellets were resuspended in 15 ml lysis buffer (50 mM NaH_2_PO_4_ pH 8.0, 300 mM NaCl and 10 mM imidazole) and sonicated on ice. The lysate was centrifuged at 16,000 rpm for 30 min at 4°C. The supernatant was then loaded onto a Ni-NTA column (Novagen) equilibrated with the lysis buffer. After washing with the washing buffer (50 mM NaH_2_PO_4_ pH 8.0, 300 mM NaCl and 25 mM imidazole) to remove unbound fractions, the elution buffer (50 mM NaH_2_PO_4_ pH 8.0, 300 mM NaCl and 250 mM imidazole) was applied. The eluate containing the recombinant protein RocF was concentrated to about 1.0 ml and then applied to a Hiload16/60 Superdex200 prep-grade gel filtration column (Amersham Biosciences) equilibrated with 20 mM Tris-HCl, pH 7.5 and 150 mM NaCl. The peak fractions corresponding to the recombinant protein were pooled and analyzed by SDS-PAGE.

The existence and concentration of divalent metal ions (Co^2+^, Ni^2+^ and Mn^2+^) in the protein were determined by flame photometry in Tsinghua University. The concentration of the protein was determined by BCA method by using BSA as a standard [26]. The oligomeric state of the enzyme was determined by gel filtration chromatography using Superdex™ 200 10/300GL.

### Arginase activity assay

The arginase assay was carried out spectrophotometrically by measuring the formation of L-ornithine at 515 nm with ninhydrin. Protein sample of apo-RocF after purification was diluted to 0.05 mg ml^−1^ with 20 mM Tris-HCl pH 7.5 and 150 mM NaCl. The sample (25 µl) was added to 25 µL of 10 mM of various divalent metal ions (final concentration of 5 mM) or 25 µl of sterile water as no metal ion negative control. The mixture were heat-activated at 55°C or were maintained on ice for 30 min. 200 µl of buffered 10 mM L-arginine (arginine buffer) was then added, and the mixtures were incubated at 37°C for 1 h. The reaction was stopped by the addition of 750 µl of acetic acid, and the color was then developed by the addition of 250 µl of ninhydrin (4 mg ml^−1^) at 95°C for 1 h. The concentration of L-ornithine was measured spectrophotometrically at 515 nm in 1.5 ml cuvettes. Representative data were normalized for protein and are presented as specific activity in nmol L-ornithine/min/mg protein ± standard deviation with a minimum of two experiments conducted in triplicate. A standard curve was generated and the slope was used to measure the concentration of L-ornithine in the reaction mixture, the method was the same as argianse activity assay, and the concentration of L-ornithine in the 1.25 ml reaction mixture was 0, 20, 40, 60, 80 and 100 µM, respectively.

### Metal ions, pH and temperature optimum

To determinate the metal ions preference of apo-RocF, the activity of the purified RocF was measured at the existence of different divalent metal ions (CoCl_2_, MnCl_2_, NiCl_2_, CuSO_4_, CaCl_2_, ZnCl_2_, MgCl_2_, FeSO_4_) at pH 3.0, 6.0 and 9.0, respectively. To determine pH optimum, buffers of different pH including citric acid (pH 3.0), sodium acetate (pH 4.0), sodium citrate tribasic dihydrate (pH 5.0), MES (pH 6.0), HEPES (pH 7.0), Tris (pH 8.0) and Capso (pH 9.0) were obtained by addition of concentrated HCl or 10 M NaOH after the addition of arginine (10 mM). To confirm the effect of heat-activation on arginase activity of apo-RocF, the purified protein was incubated with equal volume of CoCl_2_, MnCl_2_ and NiCl_2_ (25 µL) at 4°C, 37°C, 55°C and 70°C for 30 min and then used for arginase activity assay.

### Effect of reducing agents on arginase activity of apo-RocF

To determine the impact of reducing agents on arginase activity of apo-RocF, different concentrations of DTT or β-ME were added to the reaction mixture before or after incubation at 55°C at the presence of Co^2+^, while sterile water was served as positive control. The concentration of RocF, arginine, Co^2+^ and the total reaction volume were the same as previously.

### Effect of inhibitors nor-NOHA and BEC on arginase activity of apo-RocF

Apo-RocF were first heat-activated at the presence of Co^2+^, Ni^2+^ and Mn^2+^ at 55°C, arginase buffer containing different concentrations (0.1 mM, 0.5 mM, 1 mM, 5 mM and 10 mM) of two inhibitors, nor-NOHA and BEC, were then added into the reaction mixture and the inhibitory effect on arginase activity of apo-RocF was evaluated. The concentration of RocF, arginine, divalent ions and the total reaction volume were not changed, and the inhibitor free aiginine buffer was used as positive control.

## Supporting Information

Figure S1 **RocF exists as monomer in solution.** Gel-filtration chromatography (Superdex™ 200 10/300GL) for apo-RocF in 20 mM Tris-HCl, pH 7.5 and 150 mM NaCl, apo-RocF exists as monomer according to the position of the peak.
(TIF)

Figure S2 **The standard curve generated for determination the concentration of L- ornithine.**
(TIF)
