# Supplementary figures and images for: Expression, Purification and Characterization of Arginase from Helicobacter pylori in Its Apo Form
Source: PLoS One. 2011 Oct 20;6(10):e26205. doi: 10.1371/journal.pone.0026205 (PMC3197605; doi:10.1371/journal.pone.0026205)

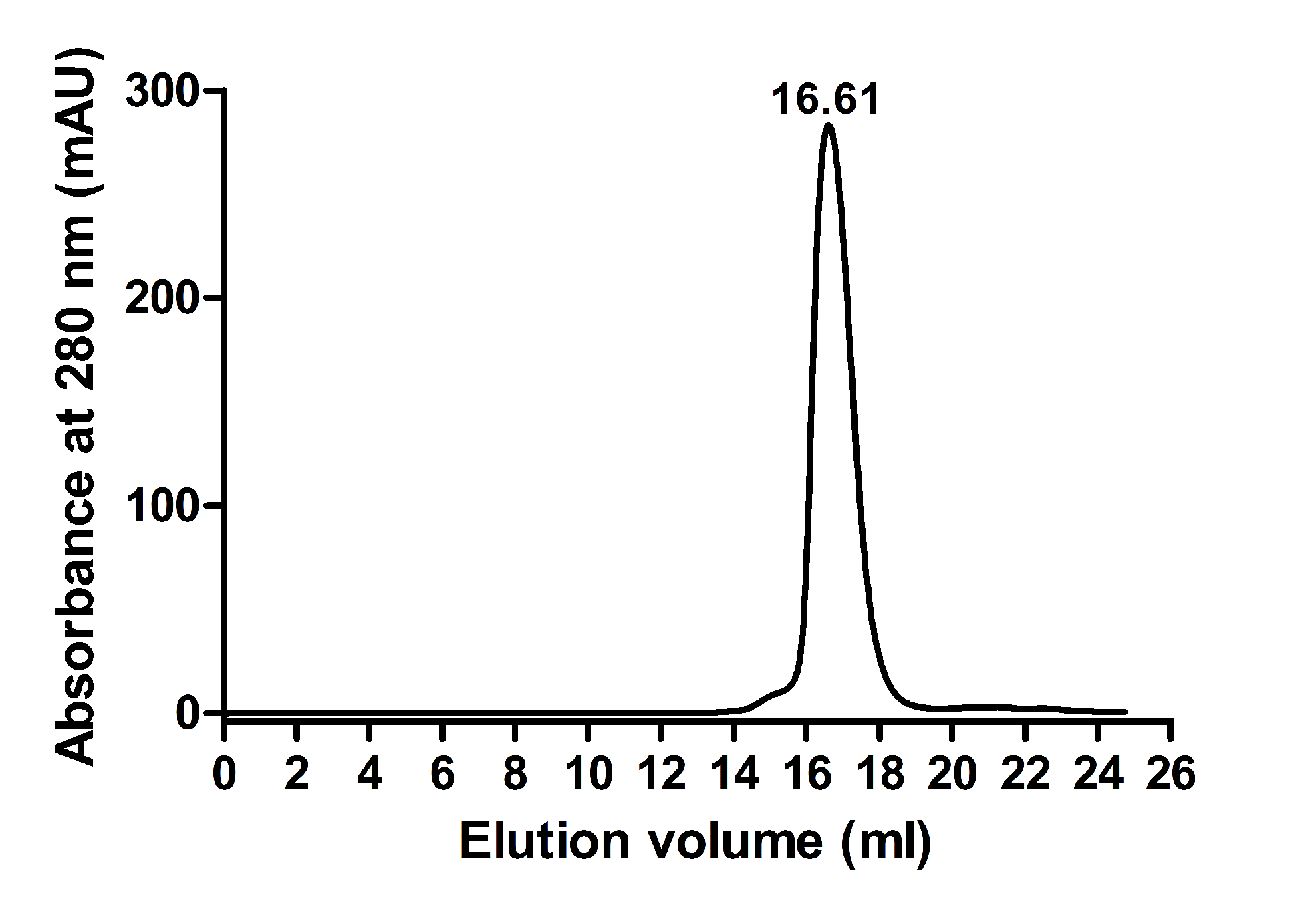

Supplement: Figure S1 — RocF exists as monomer in solution. Gel-filtration chromatography (Superdex™ 200 10/300GL) for apo-RocF in 20 mM Tris-HCl, pH 7.5 and 150 mM NaCl, apo-RocF exists as monomer according to the position of the peak. (TIF) [file pone.0026205.s001.tif]

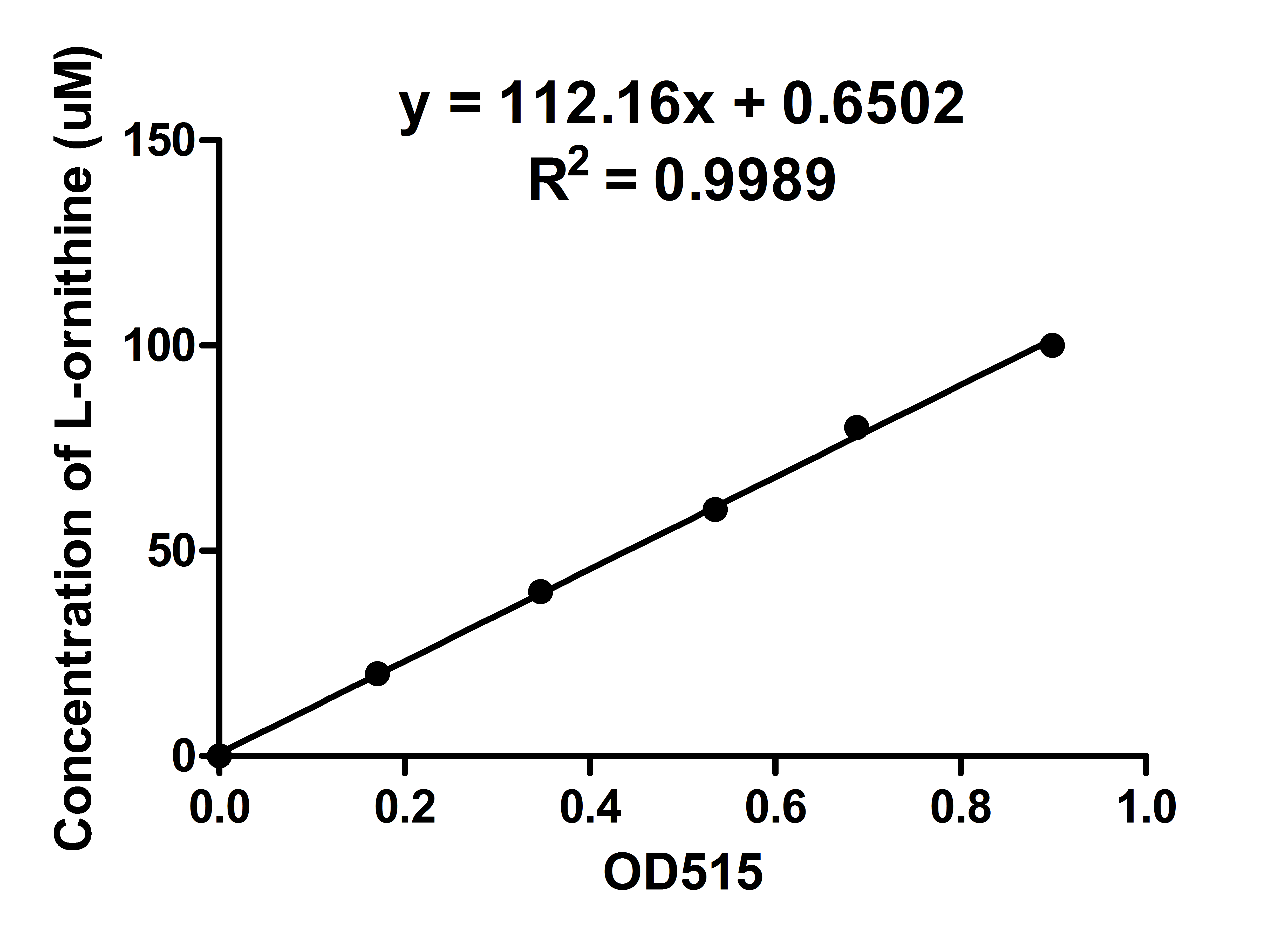

Supplement: Figure S2 — The standard curve generated for determination the concentration of L- ornithine. (TIF) [file pone.0026205.s002.tif]
